# Surgery duration: Optimized prediction and causality analysis

**DOI:** 10.1371/journal.pone.0273831

**Published:** 2022-08-29

**Authors:** Orel Babayoff, Onn Shehory, Meishar Shahoha, Ruth Sasportas, Ahuva Weiss-Meilik

**Affiliations:** 1 Bar-Ilan University, Ramat Gan, Israel; 2 I-Medata AI Center, Tel-Aviv Sourasky Medical Center, Sackler Faculty of Medicine, Tel-Aviv University, Tel Aviv, Israel; 3 Division of Medical Operation, Tel Aviv Sourasky Medical Center and Sackler Faculty of Medicine, Tel Aviv University, Tel Aviv, Israel; Al-Balqa Applied University Prince Abdullah bin Ghazi Faculty of Information Technology, JORDAN

## Abstract

Accurate estimation of duration of surgery (DOS) can lead to cost-effective utilization of surgical staff and operating rooms and decrease patients’ waiting time. In this study, we present a supervised DOS nonlinear regression prediction model whose accuracy outperforms earlier results. In addition, unlike previous studies, we identify the features that influence DOS prediction. Further, in difference from others, we study the causal relationship between the feature set and DOS. The feature sets used in prior studies included a subset of the features presented in this study. This study aimed to derive influential effectors of duration of surgery via optimized prediction and causality analysis. We implemented an array of machine learning algorithms and trained them on datasets comprising surgery-related data, to derive DOS prediction models. The datasets we acquired contain patient, surgical staff, and surgery features. The datasets comprised 23,293 surgery records of eight surgery types performed over a 10-year period in a public hospital. We have introduced new, unstudied features and combined them with features adopted from previous studies to generate a comprehensive feature set. We utilized feature importance methods to identify the influential features, and causal inference methods to identify the causal features. Our model demonstrates superior performance in comparison to DOS prediction models in the art. The performance of our DOS model in terms of the mean absolute error (MAE) was 14.9 minutes. The algorithm that derived the model with the best performance was the gradient boosted trees (GBT). We identified the 10 most influential features and the 10 most causal features. In addition, we showed that 40% of the influential features have a significant (p-value = 0.05) causal relationship with DOS. We developed a DOS prediction model whose accuracy is higher than that of prior models. This improvement is achieved via the introduction of a novel feature set on which the model was trained. Utilizing our prediction model, hospitals can improve the efficiency of surgery schedules, and by exploiting the identified causal relationship, can influence the DOS. Further, the feature importance methods we used can help explain the model’s predictions.

## Introduction

High utilization of resources such as equipment, staff, and facilities in healthcare organizations generates efficient patient flow and cuts costs [1–3]. The high cost of surgeries and operating rooms (ORs) have made them key elements for hospital administrators looking to streamline expenses [4]. OR underutilization results in negative consequences such as staff idle time, increased patient waiting times for surgeries, and more. On the other hand, OR overutilization might overload the staff, increase patient waiting time and dissatisfaction, generate disorder, increase the probability of human error, and more [3–5].

Each surgery comprises a number of procedures with a surgical staff to support it. This includes the surgeon, an anesthesiologist, nurses, and other staff members. Surgeries, roughly speaking, are either emergency or elective. The duration of surgery (DOS) is defined as the period of time during which the patient is in the OR.

DOS is the chief variable affecting surgery scheduling and OR management. Current practices in many hospitals suggest that physicians who are hospital staff members schedule the surgeries. As shown in the art, however, physicians tend to predict DOS inaccurately, thus causing sub-optimal scheduling [6]. In other hospitals, each surgery is allocated a default DOS. This default time is the computed mean duration of the specific procedures of that surgery type [2, 7, 8]. Given the suboptimality of DOS prediction and its negative effect on OR management, multiple studies developed machine learning (ML) DOS prediction models, aiming to optimize OR utilization. Those studies, however, did not examine causality and did not provide systematic explanations for the predictions derived by their models. In this research, we address these lacunae.

ML techniques are widely used in health informatics studies [9–11]. With the increase in surgery documentation in electronic health records (EHRs), ML has become very relevant for DOS prediction. Given the large size of surgery datasets and the abundance of factors that could influence DOS, ML facilitates data analysis beyond conservative factors and practices. As DOS values are continuous, ML regression models are highly appropriate for their prediction. With this in mind, we have developed a DOS regression prediction model.

Explaining predictions produced by ML models, beyond the performance metrics, is a necessary element of ML research in healthcare [12]. Understanding the importance of each feature to the model’s predictions sheds light on the model’s behavior. Such understanding allows domain experts, i.e., physicians and surgeons, to validate the model’s predictions and gives them a tool for optimizing surgery management. Methods for explaining individual predictions by the features used are known in the art. Other methods that explain the cumulative influence of features on the model’s prediction are also known. For an individual prediction, the output of such methods is the contribution of each feature to the prediction value. To calculate the cumulative feature importance, most methods average or sum the contribution of each feature across all records [13]. In our research, we utilize these methods to study the cumulative effects of features. We compute feature importance using algorithms such as Shapley Additive exPlanations (SHAP) [14, 15].

A causal relationship, unlike correlation, describes the relationship between two variables, suggesting that one has caused the other to occur [16–18]. Causal inference addresses the problem of identifying cause and effect relationships in data [17] and has a central role in the healthcare [19]. The determination that a connection between a feature and the target variable is causal indicates that intervention may be beneficial [20]. For example, one can intervene by changing the composition of the surgical staff, thus decreasing the DOS.

Earlier studies developed regression ML models to predict DOS values. Most studies used linear regression algorithms for the development of the prediction models [21–24]. Some recent studies, e.g., that by Jiao et al. (2020), employed ML algorithms, e.g., multilayer perceptron. The feature set used in those studies for model development included patient features and procedure features but did not incorporate surgical staff features as we do [23]. Additionally, their patient and procedure features comprised only a subset of those examined by our study. Further, these studies did not explain the DOS model’s predictions. Studies whose feature set is similar to ours [25, 26] developed ML models to predict length of physician appointment and length of stay in the emergency department, however, they have not analyzed DOS, nor have they generated explanations for their predictions.

Unlike previous studies, our focus is on the importance of features and the effect of that importance on the model and the predicted DOS. We study a broad range of patient features (age, gender, BMI, etc.), surgical staff features (experience, age, etc.), and surgical features. In addition, we use explanatory algorithms to analyze our model’s predictions and causal inference algorithms to study the effect of our features on DOS. Our models provide a prediction for both the elective and the emergency surgery classes. To develop our models, we cooperated with the main surgical department of one of the largest Israeli public hospitals, the Tel Aviv Sourasky Medical Center (TASM).

In addressing the challenges described above, the contribution of this study is four-fold. (1) We develop DOS prediction models whose performance levels are higher than those of existing DOS prediction models. (2) We introduce a feature set that includes novel features studied here for the first time as well as features examined by previous studies. (3) We identify the most influential features affecting DOS prediction. (4) We study the causal relationship between features and DOS via causal inference algorithms.

This study has several OR management implications. Using our prediction model, OR management teams can improve the performance of surgery scheduling in terms of patient waiting time and surgery team idle time. Using the identified causal relationships, OR management teams can control and adjust DOS values. Further, the explanatory methods elucidate the model’s predictions.

The paper proceeds as follows. The Introduction section presents the state-of-the-art, the motivation for this study, and its objectives. The Methodology section focuses on the research methodology applied according to IJMEDI checklist [27], including dataset acquisition, preprocessing, causality analysis, and feature extraction and selection. The Results section presents the empirical evaluation of our research model and discusses the results. It further compares our models to models in earlier studies. In the Discussion section, we discuss the main findings and point at future directions of our research. Finally, in the Conclusion section, we summarize the insights that were obtained in our study.

### Methodology

**Fig 1:**
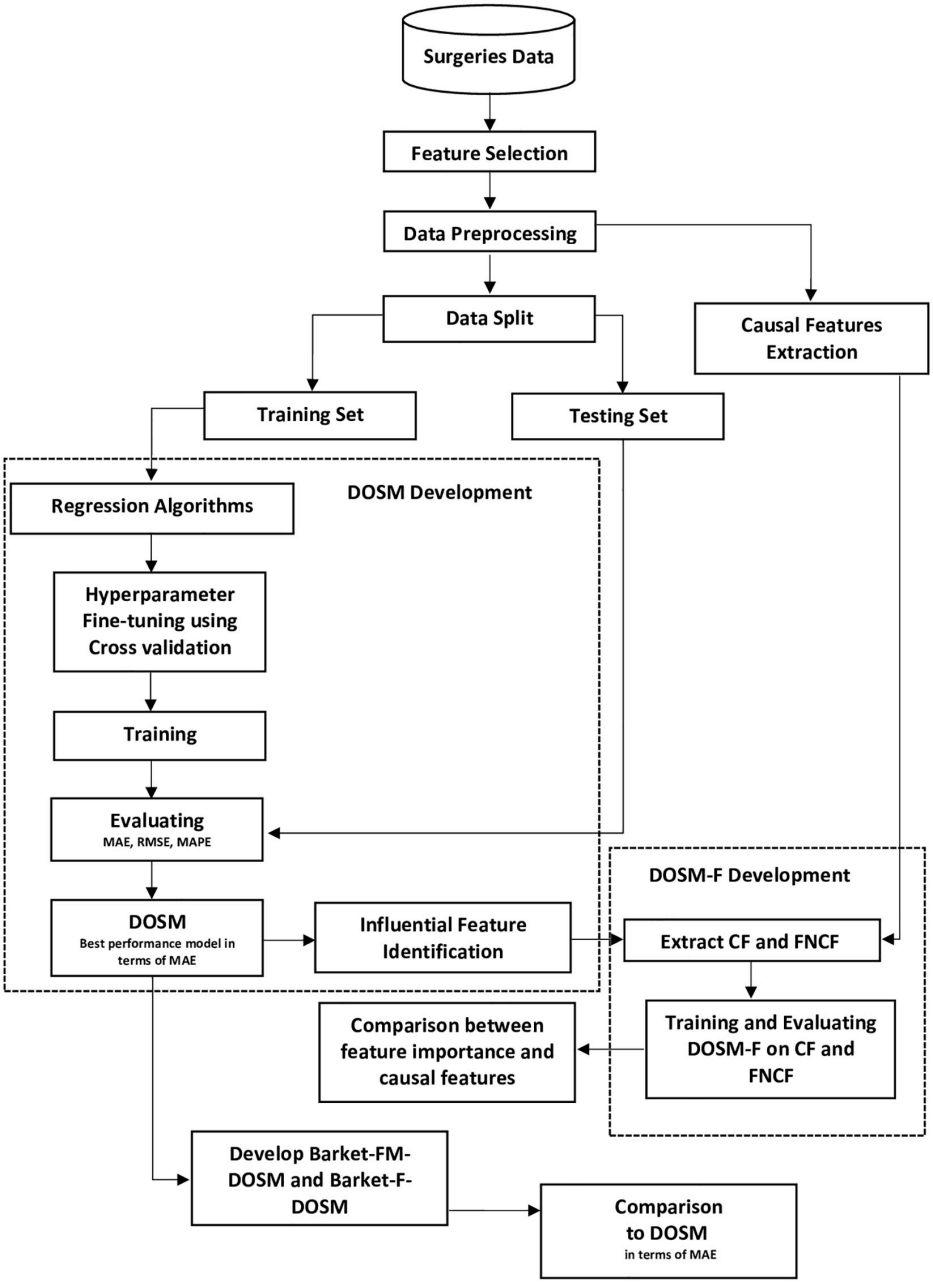
Methodology flowchart.

Our methodology comprises six stages, as follows: 1) collecting and preprocessing a dataset; 2) finding the causal relationship between features and DOS; 3) developing a DOS supervised regression model, referred to as DOSM; 4) evaluating DOSM’s performance; 5) calculating feature importance; and 6) comparing influential and causal features. Fig 1 presents a flowchart of the methodology.

### Stage 1: Data collection and preprocessing

Our surgery dataset (SD) was obtained from the Tel Aviv Sourasky Medical Center’s (TASM) (a public hospital) surgery department. The data were approved by the TASM institutional review board (IRB), approval number 0332-21-TLV. This study involves data about human participants but the IRB exempted this study from participant consent. The data were fully anonymized and then used for this study. The data included 23,293 retrospective surgical records, focusing on the eight most common surgeries in this department between 2010 and 2020. The dataset included surgical features, patient features, and surgical staff features. We examined the features that previous studies used for their DOS models and, from among them, we adopted those that are independent of the surgery type. We moreover used additional features that were suggested by domain experts, i.e., experienced surgeons [3, 8, 21, 23]. The full list of features is shown in Table 1. The table shows feature names, indication of whether a feature is novel (by a *V* in the Novel column), the value range of each feature, and values’ statistics. For numeric features, statistics include maximum, minimum, mean, and STD values. For nominal features, it includes the distribution.

**Table 1:** Description of features.

Feature	Type	Values/range and statistics	Note	Novel
**Patient features**				
Gender	Boolean	Male(55%)/Female(45%)		
Age	Numeric	Float (AVG: 47.4, STD: 19.5, MAX: 102, MIN: 18)		
Country-of-origin	Categorical	ISR (60%), ITA (0.01%), ARG (0.07%)…		
Nationality	Boolean	ISR (60%)/No-ISR (40%)		
Recent surgery types	Categorical	Chemosurgery of skin(2%), arthroscopy(4.5%), …	#1	V
Number of recent surgeries	Numeric	Integer (AVG: 0.05, STD: 2.2, MAX: 9, MIN: 0)	#2	V
Number of recent hospitalizations	Numeric	Integer (AVG: 3, STD: 4, MAX: 63, MIN: 0)	#3	V
Number of children	Numeric	Integer (AVG: 1.56, STD: 1.72, MAX: 21, MIN: 0)		V
Marital status	Categorical	Single(32%), married(50.8%), divorced(12%), widowed (5.2%)		
Chronic diseases	Categorical	Intestinal infectious diseases (001–009)(0.001%), tuberculosis (010–018) (0.001%), ….	#4	
Number of chronic diseases	Numeric	Integer (AVG: 1.42, STD:3, MAX: 11, MIN: 0)		V
Recent drugs prescribed	Categorical	Muscular pain(13%), vitamins (20%) …	#5	
Sleeping disorder	Boolean	Yes(28%)/no(72%)		V
Alcohol use disorder	Boolean	Yes(3%)/no(97%)		V
Narcotics use disorder	Boolean	Yes(1%)/no(99%)		V
Smoker/Nonsmoker	Boolean	Yes(22%)/no(88%)		
Insurance type	Categorical	Macabi (HMO-10) (24.2%), Clalit (HMO-11)(50.7%)		
BMI	Numeric	Float (AVG: 26.1, STD: 5.03, MAX: 75.6, MIN: 13.7)		
**Surgical staff features**				
Number of joint surgeries	Numeric	Integer (AVG: 50.3, STD: 14.01, MAX: 1029, MIN: 0)	#6	V
Size of the surgical team	Numeric	Integer (AVG: 6, STD: 3.2, MAX: 15, MIN: 1)		V
Number of nurses	Numeric	Integer (AVG: 2, STD: 1.9, MAX: 7, MIN: 0)		V
Number of surgeons	Numeric	Integer (AVG: 2.4, STD: 1.78, MAX: 5, MIN: 1)		V
Anesthetist gender	Boolean	Male (81%)/Female (19%)		V
Anesthetist age	Numeric	Float (AVG: 42.8, STD: 6.6, MAX: 74, MIN: 30)		V
Anesthetist country of origin	Categorical	ISR(42%), ITA…		V
Anesthetist average surgery duration	Numeric	Float: Minutes (AVG: 92, STD: 36.7, MAX: 211, MIN: 20)		V
Anesthetist experience	Numeric	Integer: Years (AVG: 3.7, STD:3.8, MAX: 34.1, MIN: 0)		
Surgeon gender	Boolean	Male (77%)/Female (23%)		V
Surgeon age	Numeric	Float (AVG: 40.9, STD: 6, MAX: 83, MIN: 26)		V
Surgeon country of origin	Categorical	ISR(45%), ITA...		V
Surgeon average surgery duration	Numeric	Float: Minutes (AVG: 92, STD: 36.7, MAX: 211, MIN: 20)		V
Surgeon experience	Numeric	Integer: Years (AVG: 4.2, STD:4.5, MAX: 38.7, MIN: 0)		
**Surgery features**				
Time slot	Categorical	Early AM (7–9 am)(31%), AM (9 am to 12 pm)(15.6%), PM (12–5 pm)(30.6%), or Night (5 pm to 7 am)(22.8%)		
Month	Categorical	Jan(9.4%), Feb(8.5%), Mar(8.8%), Apr(7.7%), May(8%)…		
Surgery room ID	Categorical	Room 1(4.2%), room 2(1.9%)…		
Surgery lead time	Numeric	Integer: Days		
Season	Categorical	Fall (26.6%), winter(24.1%), spring(22.5), summer(26.8)		
Surgery type	Categorical	Lumpectomy(13%), Perianal Abscess(6.33%), Pilonidal Sinus …		
Is private operation	Boolean	Yes (11%)/no (89%)		V
Urgency score	Ordinal	1 –Not urgent (52%), 2 (29%), 3 –urgent (19%)		V
Top 3 procedures performed	List of categorical	Codes from ICD-9		V
Surgery unit	Categorical	Unit1(37%), unit 2(9%), …		
Norton scale	Numeric	Integer: 5-20(AVG: 7, STD:8.2, MAX: 20, MIN: 5)	#7	V
Charlson comorbidity score	Numeric	Integer: 1-5(AVG: 1.1, STD:1.86, MAX: 5, MIN 1:)	#8	V
Duration	Numeric	Float: Minutes (AVG: 92, STD: 36.7, MAX: 211, MIN: 20)	#9	

^#1^ Examines how past surgeries in the 90 days preceding the current surgery affect the DOS of the current surgery; the time frame was determined by a domain expert

^#2^ The total number of surgeries in the 90 days prior to the current surgery

^#3^ Examines how past hospitalizations in the last 90 days affect DOS; the time frame was determined by a domain expert

^#4^ Examines the influence of a patient’s chronic disease categorized by The International Classification of Diseases Ninth Revision (ICD-9) with sub-chapters according to the ICD code [41]

^#5^ Drugs prescribed during the 90 days prior to the surgery, categorized by pharmacological subgroup

^#6^ The number of joint surgeries of the surgeon and anesthesiologist

^#7^ Used to identify patients at risk for pressure ulcers; a lower value indicates higher risk for pressure ulcer development [42]

^#8^ The severity of comorbidity was categorized into three bins: mild 1–2, moderate 3–4, severe ≥5 [42]

^#9^ DOS

In the data preprocessing stage, we omitted records whose DOS value was missing. We also excluded outliers, which comprised about 5% of the records. Missing surgical staff data were manually completed by the surgery department’s staff. For handling missing data of other features, we used the Sequence of Regression Models (SRM) technique for multiple inputting of missing values [28]. Accordingly, the missing values of features were computed using the values of other features.

### Stage 2: Causal inference

The causal effect of a feature on an outcome variable (in our case, DOS), e.g., in the context of medicine, is called the treatment effect or heterogeneity treatment effect (HTE) [29]. The average treatment effect (ATE) of a feature (whose value range is binary) measures the difference in the mean of the outcomes between data records with different values assigned to the feature. Since our study is observational, the ATE values could not be computed accurately, as a feature in a surgery record only has an observed value and cannot be assigned other values [29]. Consequently, we had to estimate the ATE values to measure their causal effect on DOS. Several ML algorithms are used to estimate the ATE value. For example, the ATE for a binary feature *f* is calculated as follows:

$$ATE_{f}=\frac{1}{\text{n}}\sum_{\text{i}=1}^{\text{n}}(y_{1}^{f}(\text{i})-{\text{y}}_{0}^{f}(\text{i}))$$ (1)

We use Eq (1) and its extensions to calculate the ATE. Here, $y_{1}^{f}(\text{i})$ is the value of the outcome in record number *i* when the value of feature *f* is 1. $y_{0}^{f}(\text{i})$ is the value of the outcome in record number *i* when the value of feature *f* is 0. In an observational study, $y_{1}^{f}(\text{i})$ and $y_{0}^{f}(\text{i})$ are estimated using ML algorithms. Extensions of Eq (1) that we used for calculating the ATE value of non-binary features are presented in [30].

Two main ML model types, propensity and heterogeneity models, are used for estimating causal effects. The former models are used for estimating the propensity score, which is the probability of a record to have a particular feature value given a set of observed other features, i.e., covariates. Propensity scores are used to reduce confounding variables’ effects and the implied bias. The latter models are used for estimating the heterogeneity of the treatment effect [31].

To develop the heterogeneity model, we used forest-based algorithms, which estimate non-linear HTE. The commonly used algorithms are orthogonal random forest (EstimatorDROrthoForest), forest double ML estimator, i.e., causal forest (CausalForestDML), and forest doubly robust estimator (ForestDRLearner) [32, 33]. For the development of the propensity model, we used the commonly used algorithms LassoCV, RF, and GBT [34]. To optimize the models’ hyperparameters, we used the grid search algorithm.

### Stage 3: Model development

Recent studies have shown that RF, GBT, and deep neural networks (DNNs) are capable of accurately predicting both binary and high-variance continuous variables in the healthcare domain [10, 26, 35]. Therefore, to develop the model, we utilized tree-based and DNN–based ML algorithms. Two tree-based algorithms were used, RF and GBT. One DNN-based algorithm was used–MLP.

For training and testing our model, we split the SD: 70% for training and 30% for testing. Given that the SD contained data from eight surgery types, we measured the performance metrics for the whole training set and for each of its sub-sets, partitioned by surgery type. As noted above, to optimize the model’s hyperparameters, we used the grid search algorithm. Grid search combines all possible hyperparameters to be optimized with predefined value ranges [36]. The algorithm’s output is the model’s hyperparameters whose performance levels are the highest.

### Stage 4: Model validation

To evaluate our model’s performance, we used the regression metrics Mean Absolute Error (MAE), Mean Absolute Percentage Error (MAPE) and Root Mean Square Error (RMSE). The metrics are computed as follow:

$$MAE=\frac{1}{n}\sum_{i=1}^{n}\left|y_{i}-t_{i}\right|$$ (2)

$$MAPE=\frac{1}{n}\sum_{i=1}^{n}\left|\frac{{\text{y}}_{\text{i}}-{\text{t}}_{\text{i}}}{{\text{y}}_{\text{i}}}\right|$$ (3)

$$RMSE=\sqrt{\sum_{i=1}^{n}\frac{{\text{(}y_{i}-t_{i})}^{2}}{n}}$$ (4)

Where *y*_*i*_ is the predicted DOS value of record *i*, *t*_i_ is the true value of DOS, and *n* is the number of records. To evaluate the grid search output, we used K-fold cross-validation, a commonly used method to fully and effectively utilize data [37].

To compare the performance of our model to the performance of the DOS prediction models presented in previous studies, we applied a methodology presented in a state-of-the-art study to our SD [3]. That study was selected for our comparison because the performance it achieved is better than that achieved by other studies. Further, its model’s features do not depend on a specific surgery type. One aim of this comparison was to examine whether the introduction of the novel features in our study results in better model performance than the performance of prior models. To this end, we re-implemented the model presented in [3]. The comparison was performed on the same test and training sets. The performance metric used for the comparison was MAE.

### Stage 5: Influential feature identification

To identify the features that influence the DOS prediction, we employed feature importance methods that do not depend on the algorithm type. First, we utilized Pearson correlation to compute the correlation between the independent features and the dependent feature. Then, we used SHAP to estimate the contribution of each feature to the model’s prediction [38, 39].

### Stage 6: Comparison between influential and causal features

We compared the influential features and the causal features. To this end, we filtered out features that had high correlation with the causal features so that the comparison would not be based on highly correlated features. To filter, we initially split the feature set F into two subsets. The first set–causal feature set (CF)–includes features whose absolute ATE value is greater than 0 and are identified as significant causal features (using *P* = .05). The second subset–non-causal feature set (NCF), NCF = F \ CF–includes the remaining features. The filtering process was done by calculating the Pearson correlation between the causal features in CF and the non-causal features in NCF and omitting NCF features that highly correlate with CF features (i.e., the correlation value is greater than 0.49) [40]. The resultant filtered subset, whose member features are NCF features that are not correlated to CF features, is the filtered non-causal feature set (FNCF).

To calculate feature importance, we developed a DOS prediction model using the features in CF and FNCF. We call this model DOSM-F, as it is similar to DOSM, but with filtered features. For the comparison we used the influential features of DOSM-F and the causal features. We aimed to identify features that influence DOS prediction and also have a causal relationship with DOS. In addition, we examined whether a feature that has a positive causal effect on DOS also has a positive effect on the DOS predicted value, and whether a feature that has a negative causal effect on DOS also has a negative effect on the DOS predicted value.

The DOSM-F model was used to estimate the potential change in the DOS as a result of variations in causal feature values (for example, potential changes in the surgical staff size). We used DOSM-F because the features used for training that model were CF and FNCF. Training using only these features allowed the CF values to have a bigger impact on the prediction value of DOSM-F compared to the prediction value derived when using all features including the features correlated with CF.

## Results

### Implementation

DOSM development and data analysis were done via Python scripts using the EconML [43], scikit-learn, LightGBM, NumPy, SHAP, and scikit-feature packages.

### Data analysis

Table 2 summarizes the statistical metrics of DOS in minutes across the surgery types, without our model being applied. Patients’ average age of is 47.5 years and the STD is 19.5.

**Table 2:** Dataset statistics.

Surgery type	Mean DOS	STD DOS	Dataset size
Laparoscopic Cholecystectomy	117	38.9	5,648
Repair of Inguinal Hernia Laparoscopic	85.5	26.8	5,240
Laparoscopic Appendectomy	98	23.7	4,902
Lumpectomy	113.5	36	3,023
Perianal Abscess	38.3	11.9	1,475
Pilonidal Sinus	76.3	15	1,142
Anal Fistulotomy	47.3	13.7	943
Excision of Hemorrhoids	57.7	12.8	920

**Fig 2:**
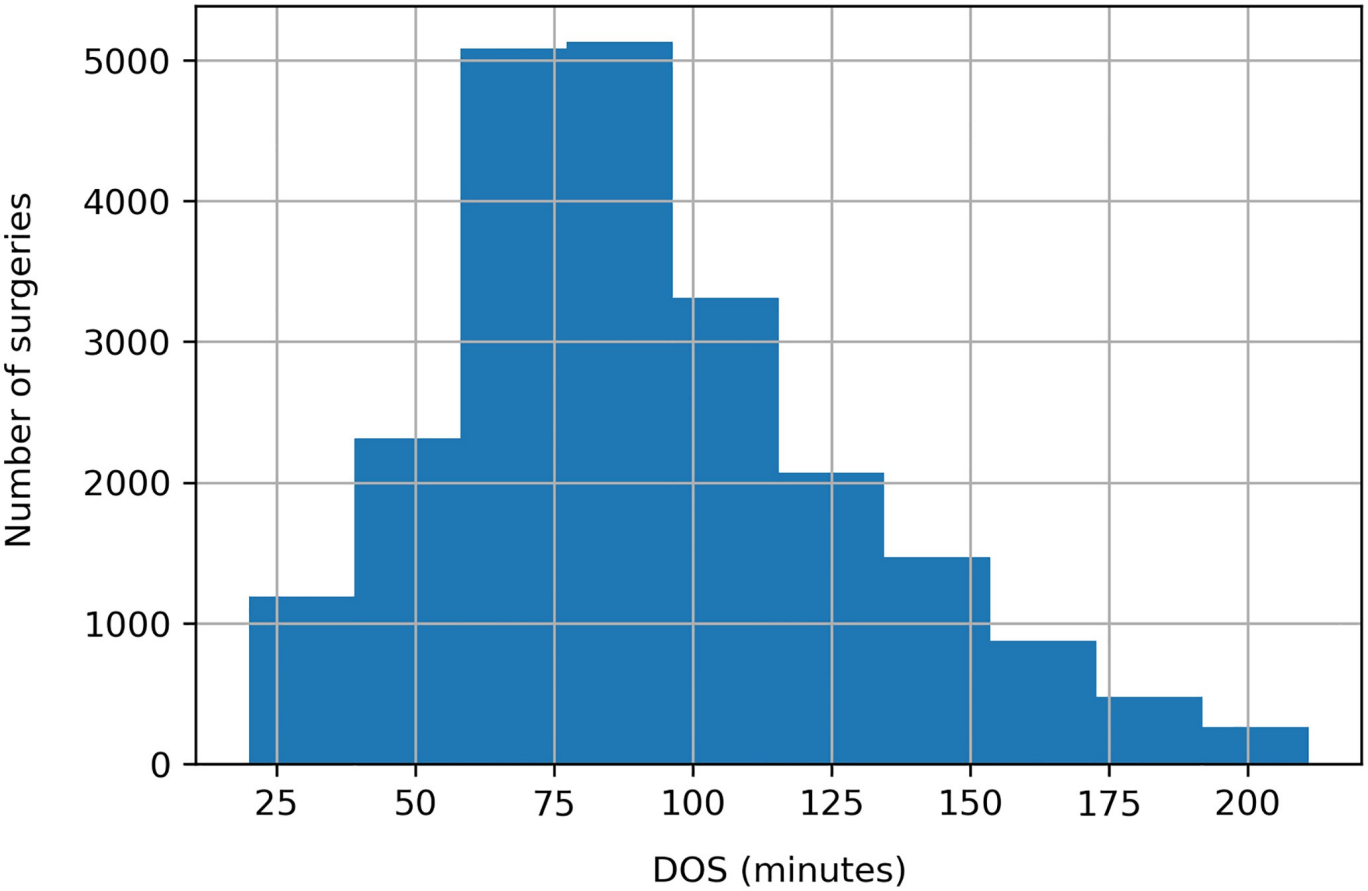
DOS distribution.

Fig 2 presents the DOS distribution, which is a positively skewed distribution. The high STD values across the surgery types indicate that the regression predicted values, i.e., the predicted DOS values, are spread over a broad range.

### Causal inference

#### Causal model development

The causal analysis models we used were trained on the SD. The inputs to these models are a vector of the counterfactual features X, a vector of the outcome feature Y (i.e., the model’s target feature), and a vector of a selected feature T–a candidate causal feature. The SD comprises *n* feature column vectors *f*_*i*_ and one target feature *Y*, i.e., *SD = {f*_*1*_,*…*, *f*_*n*_, *Y}*. To extract X, Y and T from the SD, we select *T = f*_*i*_, *X* = *SD*\{*f*_*i*_, *Y*}; Y is the DOS column of the SD. We iterated over *i* and calculated the ATE for all *f*_*i*_ features. Thus, we obtained the causal effect of all the features on DOS.

#### Hyperparameter optimization

The hyperparameter values we used to optimize the HTE and propensity models are listed in Table 3. The LassoCV algorithm is an iterative algorithm that finds the optimal parameters for a Lasso model using cross-validation; thus, this algorithm does not appear in Table 3 [44]. The hyperparameter values used (see Table 3) are similar to values commonly used in the art.

**Table 3:** Causal inference models’ hyperparameter values.

Model	Algorithm	Hyperparameter	Values
Propensity model	GBT	Number of trees	50, 100, 150, 300, 600, 1200, 2400
		Learning rate	0.001, 0.005, 0.01, 0.025, 0.05, 0.1, 0.2, 0.5
		Maximum depth of a tree	10, 20, 40, 80, 160
		Maximum tree leaves	3, 7, 31, 100, 500, 1000, 2000
Propensity model	RF	Minimum number of samples required to split a node	2, 5, 10, 20
		Number of trees	50, 100, 150, 300,600, 1200, 2400
		Minimum number of samples required at each leaf node	1, 2, 5, 10, 20, 40
		Maximum depth of a tree	10, 20, 40, 80, 160
		Maximum features in a tree	unlimited, log2, sqrt
HTE model	EstimatorDROrthoForest, CausalForestDML, ForestDRLearner	Minimum weighted fraction of the sum total of weights	0.0001,0.001, 0.01, 0.1
		Maximum depth of the tree	3, 6,10, 20, 40, 80
		Minimum variation of the treatment vector that should be contained within each leaf	0.0001,0.001, 0.01, 0.1
		Maximum features in a tree	unlimited, log2, sqrt

#### Causal feature identification

Table 4 presents the 10 features whose absolute ATE values were the highest, in decreasing order. Six of the 10 most causal features are also among the 10 most correlated features by Pearson correlation (shown in Table 9). Half of the top 10 causal features are among the novel features shown in Table 1.

**Table 4:** ATE values.

	Feature	ATE
**1**	Surgery type	-15
**2**	Procedure 1 code	-10
**3**	Surgeon–average surgery duration	8.8
**4**	Surgery urgency score	7
**5**	Patient–alcohol use disorder	-6.7
**6**	Patient–recent drugs prescribe	6
**7**	Patient–number of chronic diseases	-5.5
**8**	Surgery time slot	-3.8
**9**	Patient–gender	2.9
**10**	Patient–country-of-origin	2.8

### Model development

#### Hyperparameter optimization

The hyperparameter values we used for optimizing our DOS prediction models appear in Table 5. These values are similar to values commonly used in the art [45–49].

**Table 5:** Hyperparameter values.

Algorithm	Hyperparameter	Values
GBT	Number of trees	50, 100, 150, 300, 600, 1200, 2400
	Learning rate	0.001, 0.005, 0.01, 0.025, 0.05, 0.1, 0.2, 0.5
	Maximum depth of a tree	10, 20, 40, 80, 160
	Maximum tree leaves	3, 7, 31, 100, 500, 1000, 2000
RF	Minimum number of samples required to split a node	2, 5, 10, 20
	Number of trees	50, 100, 150, 300,600, 1200, 2400
	Minimum number of samples required at each leaf node	1, 2, 5, 10, 20, 40
	Maximum depth of a tree	10, 20, 40, 80, 160
	Maximum features in a tree	auto, log2, sqrt
MLP	Number of hidden layers	1, 2, 4, 8, 16
	Number of nodes in each hidden layer	4, 8, 16, 32, 64
	Number of epochs	50, 100, 200, 400, 800, 1600
	Learning rate	0.001, 0.005, 0.01, 0.025, 0.05, 0.1, 0.2, 0.5
	Activation function	Relu, Tanh
	Solver	SGD, ADAM

#### Model training and performance

We trained the DOS models on the dataset using several ML algorithms. The ML algorithms calculate the features’ influence differently; for this reason, the models were trained on all of the features. The algorithms that generated the top performing models–GBT being the best–are presented in Table 6. Overall, the MAE values in the table suggest that the performance is similar across the three algorithms, with GBT performing a bit better. Table 7 presents the per surgery type performance of the GBT model. This was done by splitting the test set by surgery type. In addition, to evaluate the effectiveness of our model against current practices, we calculated the MAE value of the manual method currently used by the clinics’ staff for each surgery type. In the manual method, the mean of the previous surgery by type is used to estimate the future DOS. The DOSM performance is significantly better than that of the manual method (using *P* = .05) (see Table 7).

**Table 6:** DOS models’ performance (in minutes).

Algorithm	MAE	RMSE	MAPE
MLP	15	20.5	0.172
GBT	14.9	20.3	0.164
RF	15.9	21.7	0.181

**Table 7:** GBT model performance by surgery type in terms of MAE (in minutes).

Surgery type	MAE	MAE manual method
Laparoscopic Cholecystectomy	17.9	32.6
Repair of Inguinal Hernia Laparoscopic	14.8	21.4
Laparoscopic Appendectomy	14.5	19
Lumpectomy	19	30
Perianal Abscess	7.4	9.5
Pilonidal Sinus	10.4	12.2
Anal Fistulotomy	11	12
Excision of Hemorrhoids	8.5	10.5

We have calculated the model’s uncertainty as follows. First, for each record in the test set, we used the DOSM to predict a list of probabilities from each tree in the GBT. Then, for each record, we calculated the STD from the list of probabilities. Finally, we calculated the mean of the STDs. Following this flow, the derived uncertainty of the model was 4.1 minutes.

### Comparison to recent results

To compare our model’s performance and examine whether the novel features introduced in our study derive a model that outperforms the state of the art, we developed two additional models. The first one, Barket-FM-DOSM, is a DOS model using the features and the methods used in Barket et al. (2019), but trained on our SD. The second one, Barket-F-DOSM, is a DOS model taking only the features used in Barket et al. (2019), but using our methods and trained on SD.

The results of the comparison are shown in Table 8. One can observe that the MAE value of our model–DOSM–is lower than the MAE values derived for Barket-F-DOSM and Barket-FM-DOSM, indicating that our model outperforms recent models, presented in Barket et al. (2019). This comparison led to the conclusion that neither the ML algorithms nor the dataset are the source of differences in the models performance. The major effector of such differences is the set of features.

**Table 8:** Comparison between models by MAE value (in minutes).

Model	MAE
DOSM	14.9
Barket-F-DOSM	16.4
Barket-FM-DOSM	18.8

### Feature importance

**Fig 3:**
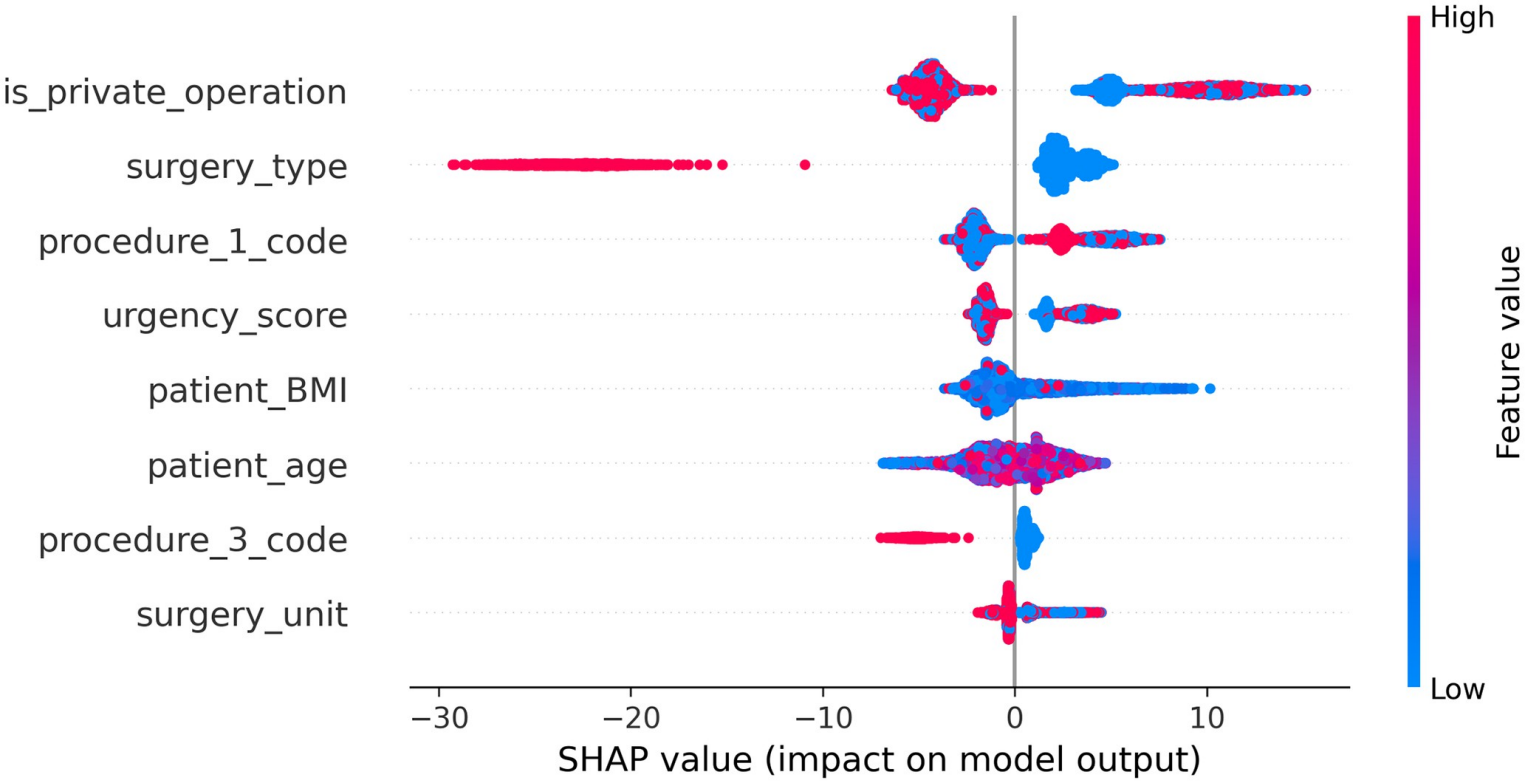
SHAP—DOSM.

Feature importance was computed using the SHAP algorithm. SHAP computes importance values for all features. To select the most influential features, we transformed the importance values distribution into a normal distribution (via a log transformation). From that normal distribution, we selected only the features whose values were one standard deviation from the rightmost edge of the distribution. The features left were selected as the most influential ones. Fig 3 illustrates the 8 most influential features on DOS prediction computed by SHAP, in a decreasing order of importance. The higher the vertical location–the higher the feature’s importance. Each point in Fig 3 is a SHAP value of a record per feature. The latter determines its position on the y-axis and the former (the record), its position on the x-axis. The color represents the value of the feature from low to high; red indicates that the feature’s value is high. Overlapping points are jittered in the y-axis direction. The horizontal location of a dot indicates its feature’s value effect on DOS, i.e., the impact on the model’s output. Half of the 10 most influential features are among the novel features presented in Table 1 in Section 2.

In Table 9, the 8 features with the highest absolute Pearson correlation values vis-à-vis DOS are presented in decreasing order of correlation values. Features whose correlation values are smaller than 0.3, which are considered weak according to common practices [50], are not presented.

**Table 9:** Pearson correlation values.

	Feature	Correlation
**1**	Patient–recent drugs prescribe	0.52
**2**	Patient–country-of-origin	0.5
**3**	Surgery type	0.48
**4**	Procedure 1 code	0.46
**5**	Procedure 3 code	0.44
**6**	Surgery room ID	0.4
**7**	Urgency score	0.36
**8**	Surgery Norton scale	0.31

From the above results, we observe that 3 out of 8 (37.5%) of the features selected are the same for both methods, SHAP and Pearson correlation.

### Comparison between feature importance and causal features

**Fig 4:**
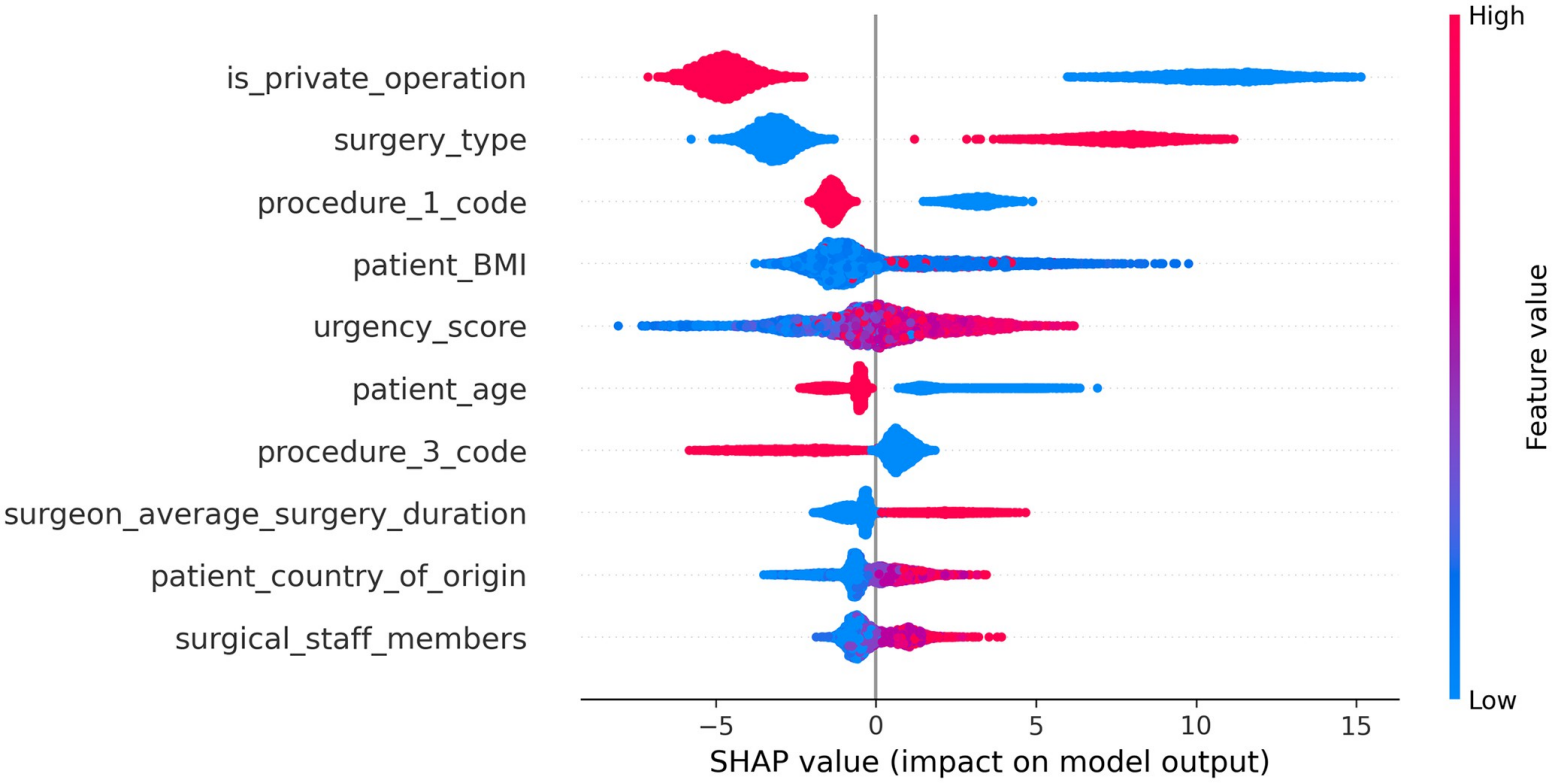
SHAP—DOSFM.

For the development of DOSM-F we used the same algorithms and hyperparameters used for developing DOSM. The performance of DOSM-F was 15.4 minutes in terms of MAE. The 10 most influential features are presented in Fig 4.

**Fig 5:**
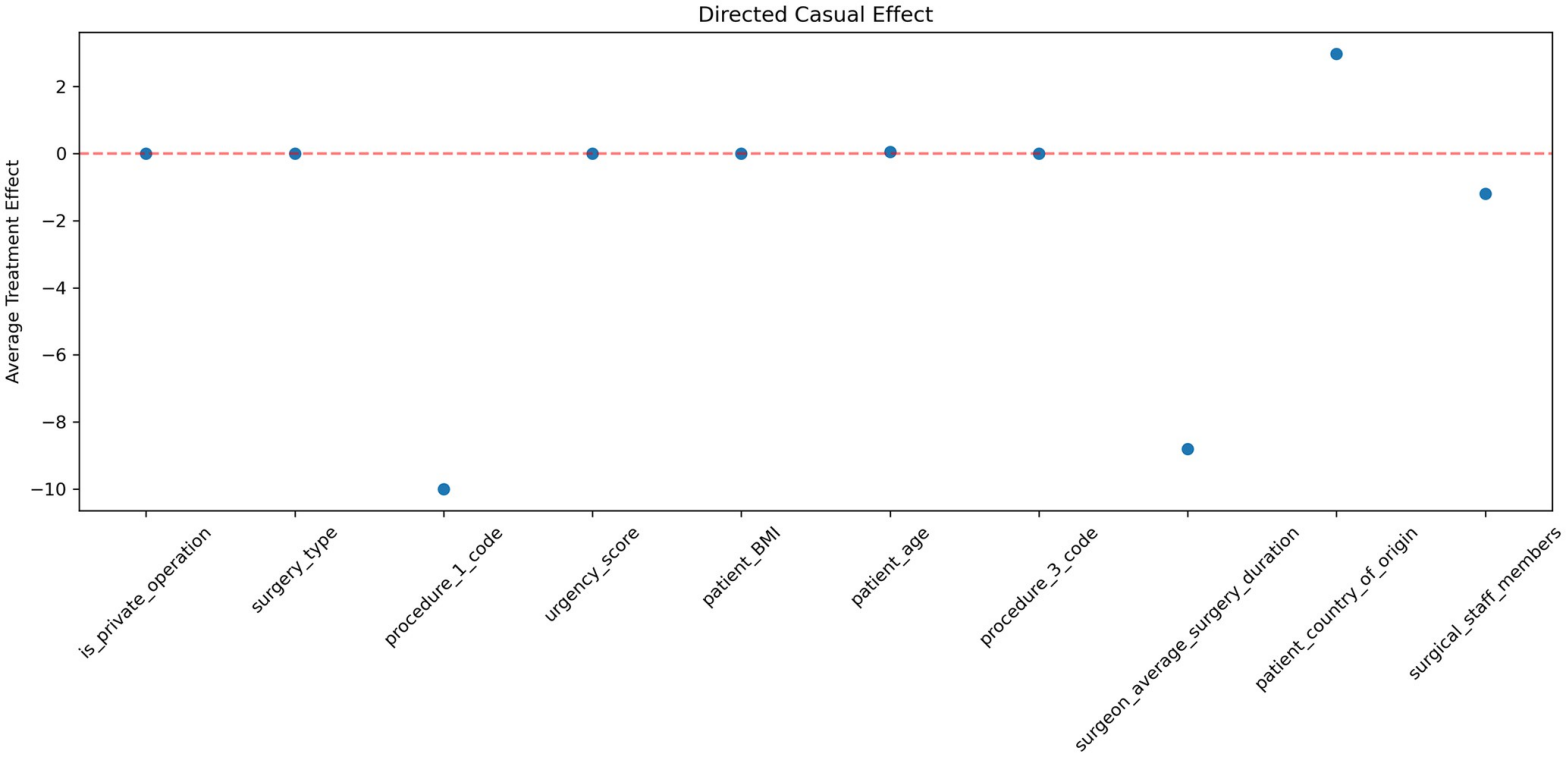
The ATE value of the 10 most influential features by SHAP.

Fig 5 presents the influential features’ causal relation to DOS by showing the ATE value of the 10 most influential features ordered by their importance in the same order as in Fig 4. It demonstrates that the order of the 10 most important features by influence and by causal value is different. 40% of the important features have a significant (*P* = .05) causal relationship with DOS. Our results reinforce the assertion made in the art that the features that influence prediction are not necessarily causal features [51]. Figs 4 and 5 show that an influential feature that has a positive effect on the predicted DOS value also has a positive causal effect on DOS and vice-versa.

## Discussion

Surgeries are one of hospitals’ largest expenditure sources. Hence, optimizing their flow to reduce costs is an important objective. Improving resource utilization, minimizing surgery lead time, and minimizing patient waiting time in the waiting room could help achieve this goal. This study presented methods to facilitate such optimization.

We utilized ML techniques to develop supervised ML models that predict DOS from features related to patients, physicians, and surgeries. For training the models, we built a dataset of 23,293 records, collected and processed in collaboration with one of the biggest public hospitals in Israel. Our dataset contained data on eight types of surgeries from the years 2010 to 2020. Our feature set combines novel features used for the first time here and features adopted from previous studies.

The performance of our DOS model in terms of MAE was 14.9 minutes. The ML algorithm that derived the model with the best performance was the GBT. We compared the performance of our model to the performance of existing models by re-implementing the latter and training them on our dataset. Our model outperformed earlier models.

The main goal of this study was to identify the features that were most influential in predicting DOS and the features that have a causal relationship with DOS. To this end, we utilized feature importance methods to identify the influential features, and causal inference methods to identify the causal features.

We demonstrated that five of the 10 most influential features on DOS prediction and five of the 10 most causal features on DOS are among the novel features we introduced in this study. In addition, we have shown that most of the influential features do not have a causal relationship with DOS.

The results of this research have several implications. Firstly, using the DOS value predicted by our model for surgery scheduling can decrease patient waiting time and minimize surgical staff idle time. Additionally, using the identified causal relationship, OR management teams can apply measures to affect DOS. This can be done, for example, using the DOSM-F model and estimating the potential change in DOS as a result of variations in causal feature values. Further, the explanatory methods we used can facilitate validation of the model’s prediction.

There are some limitations in our study. Our datasets contained data of eight surgery types. Future research could study additional surgery types at different hospitals to broaden applicability of our results. A future study can evaluate the performance of the prediction model when combined with a scheduling system in a production environment. Further research is needed to quantify the potential cost-saving and OR utilization when using the DOSM.

## Conclusions

We used ML methods to develop a supervised regression model to predict DOS using various novel features of patients, surgical staff, and surgeries. The model we developed outperformed the current method used in hospitals and the DOS models developed in previous studies. Several insights were obtained in our study, including identification of the most influential features on DOS prediction and identification of the causal relationship between the features and DOS.
